# Contribution of Wastewater Irrigation to Soil Transmitted Helminths Infection among Vegetable Farmers in Kumasi, Ghana

**DOI:** 10.1371/journal.pntd.0005161

**Published:** 2016-12-06

**Authors:** Isaac Dennis Amoah, Amina Abubakari, Thor Axel Stenström, Robert Clement Abaidoo, Razak Seidu

**Affiliations:** 1 Institute for Water and Wastewater Technology, Durban University of Technology, Durban, South Africa; 2 Department of Civil Engineering, Norwegian University of Science and Technology, Ålesund, Norway; 3 Department of Theoretical and Applied Biology, Kwame Nkrumah University of Science and Technology, Kumasi, Ghana; Hospital General, MEXICO

## Abstract

Wastewater irrigation is associated with several benefits but can also lead to significant health risks. The health risk for contracting infections from Soil Transmitted Helminths (STHs) among farmers has mainly been assessed indirectly through measured quantities in the wastewater or on the crops alone and only on a limited scale through epidemiological assessments. In this study we broadened the concept of infection risks in the exposure assessments by measurements of the concentration of STHs both in wastewater used for irrigation and the soil, as well as the actual load of STHs ova in the stool of farmers and their family members (165 and 127 in the wet and dry seasons respectively) and a control group of non-farmers (100 and 52 in the wet and dry seasons, respectively). Odds ratios were calculated for exposure and non-exposure to wastewater irrigation. The results obtained indicate positive correlation between STH concentrations in irrigation water/soil and STHs ova as measured in the stool of the exposed farmer population. The correlations are based on reinfection during a 3 months period after prior confirmed deworming. Farmers and family members exposed to irrigation water were three times more likely as compared to the control group of non-farmers to be infected with *Ascaris* (OR = 3.9, 95% CI, 1.15–13.86) and hookworm (OR = 3.07, 95% CI, 0.87–10.82). This study therefore contributes to the evidence-based conclusion that wastewater irrigation contributes to a higher incidence of STHs infection for farmers exposed annually, with higher odds of infection in the wet season.

## Author Summary

Wastewater irrigation in agriculture is a common reality in many developing cities, linked to rapid urbanization. Approximately 50%-90% of urban dwellers in West Africa consume wastewater/ polluted surface water irrigated-vegetables within cities with 10% of the population involved in the practice. Viral, bacterial and parasitic pathogens can all be found in wastewater putting exposed populations at risk of pathogenic infections. The biggest risk however is to helminth infections, due to their long survival time in the environment. Wastewater irrigation has been practiced in Ghana for many years, however few studies have investigated the epidemiological link between the practice and helminth infections among the farmers. In this study the authors measured the helminth ova concentration in wastewater used for irrigation and the infection loads of farmers as well as a non-farmer control group in Ghana. They reported high concentrations of helminth ova in the wastewater as well as soil on the farms, above the World Health Organization (WHO) guidelines for wastewater irrigation, which resulted in a three times higher probability of infections with helminths for farmers as compared to the non-farmer control group. This research provides information on the direct link between wastewater irrigation and helminth infection in exposed individuals.

## Introduction

Wastewater use in agriculture has been promoted as part of the concept of sustainable development. In many cities in developing countries, wastewater irrigation is a common reality linked to rapid urbanization. The practice improves farmers’ livelihoods, contributes to the urban food basket and slightly improves the urban environment by diverting wastewater to agricultural fields [1]. In Sub-Saharan Africa (SSA), it is estimated that 10% of the population in cities are involved in the practice of wastewater irrigation, with 50% to 90% of urban dwellers in West Africa reported to consume vegetables irrigated with wastewater or polluted surface water within or close to cities [2]. In Ghana, a significant proportion of untreated wastewater is discharged into drains and nearby water bodies, which is then used by farmers for irrigation. Wastewater irrigation in the cities of Ghana is mainly for the production of vegetables, such as cabbage, lettuce, spring onion and carrots [3].

Although there are many benefits associated with wastewater irrigation, the practice can lead to significant health risk if not undertaken in a safe manner [4]. All enteric pathogens of viral, bacterial and parasitic (helminthic and protozoan) origins can be found in wastewater; and can be transmitted to farmers using the wastewater for irrigation, consumers of wastewater-irrigated vegetables and communities close to wastewater irrigated fields [5]. Several studies have shown a significant relationship between *Ascaris* infection and exposure to wastewater (either treated or untreated) [6,7,8]. This is because soil transmitted helminths (STHs) (such as *Ascaris*) can survive for long periods of time under severe adverse environmental conditions [9] contributing to their high risk of infection. STHs are common worldwide with more than a billion people infected [10, 11]. Estimates suggest that *Ascaris lumbricoides* infects over 1 billion people, *Trichuris trichiura* 795 million, and hookworms (*Ancylostoma duodenale* and *Necator americanus*) 740 million [12]. Farmers in Pakistan using wastewater for irrigation have been reported to be five times more likely to be infected with hookworms than others using canal water [13] and in Dakar, Senegal the reported incidence of amoebiasis and ascariasis is 60% in farmers involved in wastewater irrigation [14]. In a study in Mexico, irrigation with untreated or partially treated wastewater was directly responsible for 80% of all *A*. *lumbricoides* infections and 30% of diarrheal disease in farm-workers and their families [15]. The health risk can differ depending on age and gender. An epidemiological study by Habbari *et al*. [16] undertaken in Morocco to determine possible health risks associated with raw wastewater use in agriculture found ascariasis infection to be approximately five times higher, especially in children in wastewater impacted regions compared to control regions. In another study Fuhrimann *et al* [35] found that farmers exposed to wastewater in Uganda were more likely to be infected with helminths than slum dwellers and workers involved in sludge collection. However, in Vietnam, Trang *et al*. [17] found no evidence that rice cultivation with wastewater posed any significant helminth infection risk to farmers, even though they were exposed to wastewater containing 40–200 helminth eggs/L. Prevalence of and risk factors for helminth infections have been studied in Ghana [18]. Although wastewater irrigation has been the practice for many years in Ghana, especially in major cities (e.g. Accra, Kumasi, Tamale), there are no studies that have investigated the epidemiological link between the practice and helminth infections among the farmers. Studies in Ghana have reported a mean helminth ova concentration of 5–10 helminth ova per liter in water used for irrigation by farmers [19,20].

In this regard this study aimed at determining the association between STHs ova concentration in wastewater used for irrigation as well as in farm soil that farmers are exposed to and the actual infection loads in order to ascertain the epidemiological link between wastewater irrigation and risk of STHs infection for farmers. The aim of this study was achieved as it was deduced that farmers had a higher probability of infection than non-farmers.

## Methods

### Study Area

**Fig 1:**
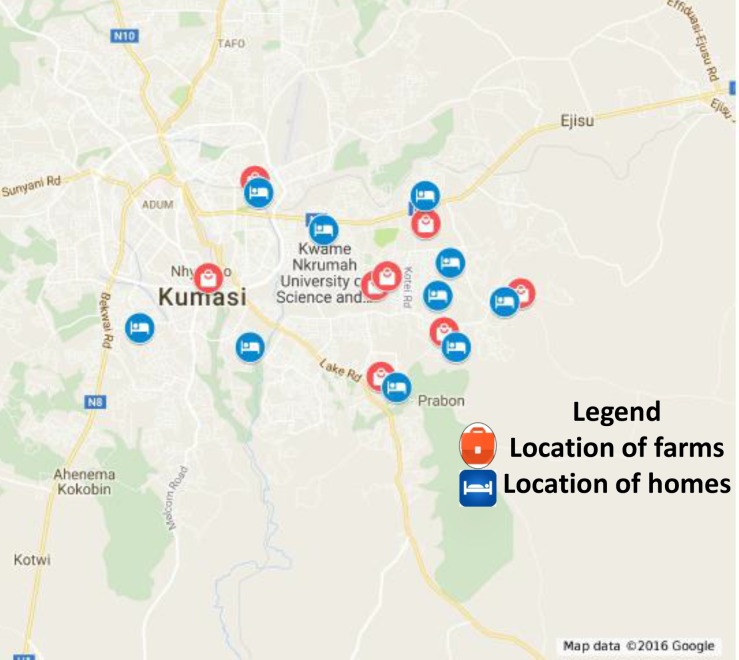
A map of Kumasi showing the location of the wastewater farms and homes of the farmers and control (courtesy Google Maps).

The study was conducted in wastewater irrigated vegetable farms in the Kumasi Metropolitan Area of Ghana (Fig 1). The Metropolis has two major seasons, the rainy (April to October) and the dry one (November to March). Relative humidity ranges from 60–84% with daily minimum and maximum temperatures of 21.5°C and 30.7°C, respectively [21]. The majority of vegetable farms in Kumasi are irrigated with wastewater which is most predominant in the dry season. Wastewater from domestic and small-scale industrial (e.g vehicle garages, saw mills, welding shops, tanneries etc) sources are discharged directly into stormwater drains and streams and collected for irrigation by farmers.

### Inclusion and Exclusion Criteria

An initial survey was carried out in the Kumasi Metropolis to identify the farms using wastewater for irrigation. This included a detailed explanation of the purpose of the study and farmers and control-group who gave consent to be part of the study were recruited. Non-farmer (control group) inhabited the same areas as the farmers and recruitment was made concomitantly. The control group thereby constituted members of families of their communities who did not take part in the practice of wastewater irrigation but stayed in the same neighborhood (as can be seen in Fig 1 below). An initial prevalence survey was undertaken after which participants were dewormed and the efficiency of the deworming exercise assessed directly afterwards. The farmer group consisted of 165 (in the wet season) farmers and family members dropping to 127 in the dry season, while the control group originally consisted of 100 individuals (in the wet season), dropping to 52 in the dry season.

The exclusion criteria was arrived at after the administration of the questionnaires to all participants, afterwards any participant who did not fall within the criteria set (based on self-reporting) was not included in the final data used for analysis, hence the dropout rate, but the project team still visited them and administered antihelminthic drugs when needed so as to encourage participation in subsequent studies.

Wastewater farmers recruited into the cohort met the following inclusion criteria: a) did not consume vegetable salad irrigated with wastewater from their farms; b) used improved toilet facilities at home and at work; c) did not use protective clothing during farm work; and d) had access to treated drinking water in their homes/communities.

### Ethics Statement

The Committee on Human Research, Publications and Ethics (CHRPE) of the Kwame Nkrumah University of Science and Technology (KNUST) approved the study (No. CHRPE/RC/051/12) with additional informed oral consent received from all participants. Informed oral consent of parents or guardians was received for all children who participated in the study, which was written on the field questionnaire administered to each person. The purpose and details of the study was explained to all participants in Twi (a local dialect) in the presence of a witness and those willing to participate gave their consent, which was noted on the questionnaires. Each participant was given a unique identifier which was used throughout the study for confidentiality. After the initial deworming exercise all participants who became re-infected were treated again with 400 mg of albendazole (XL Laboratories PVT Ltd).

### Sampling

Irrigation water was collected from August 2012 to October 2012 to represent the wet season and December 2012 to March 2013 to represent the dry season. Irrigation water samples were taken from storm drains, streams, shallow wells and potable water pipes (in a few instances), which represented the sources of water in use by the farmers. Soil samples were taken from the vegetable beds that were irrigated with the water types sampled as stated above. In all, 214 and 156 samples (for soil and irrigation water) were taken during the wet and dry seasons, respectively. All samples were collected in the morning between 06.00 and 10.00 (Greenwich Mean Time (GMT) on each day of sampling. Irrigation water and soil samples were collected in triplicates into sterile pre-labeled sample bottles (about 4 L for the wastewater) and plastic re-sealable bags (30 g composite soil sample each) and kept in a cooling box and transported to the laboratory where they were processed and analyzed for helminth eggs using the Modified EPA Method [22]. The helminths eggs were identified on the basis of their shape and size with the aid of bench aids for the Diagnosis of Intestinal Parasites [23]. Only viable helminth eggs were counted, viability was assessed based on the presence of motile larvae within the eggs. Stool samples were collected from farmers and family members as well as the non-farmer control group and analyzed using the formal-ether concentration method [23]. After three months, stool samples were taken again from the participants for assessment of re-infections.

### Statistical Analysis

Descriptive analysis was undertaken to assess the mean concentration and distribution of ova in the irrigation water and soil and described by box plots (Stata, Statacorp, Texas, USA). Analysis of variance was performed to determine the statistical difference between the concentrations of *Ascaris* spp and hookworm in the dry and wet seasons. The relationship between STHs loads in irrigation water/soil and actual STHs ova per gram of faecal matter from the farmers was determined using Poisson regression analysis (Stata, Statacorp, Texas, USA). The odds ratio (OR), its standard error and 95% confidence intervals were calculated according to Altman [24].

## Results

### STHs Ova Concentration in Irrigation Water and Soil

**Fig 2:**
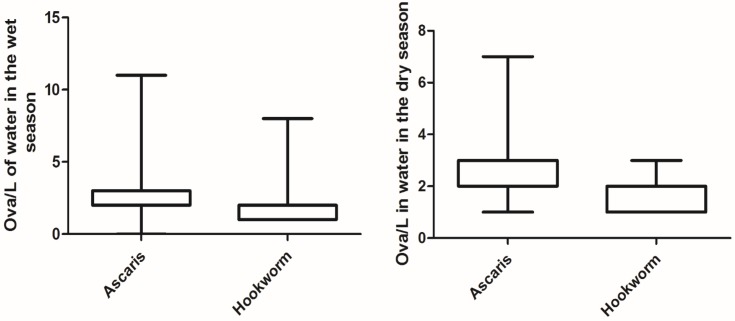
Distribution of *Ascaris* spp and hookworm ova in irrigation water in the dry (n = 71) and wet (n = 107) seasons.

**Fig 3:**
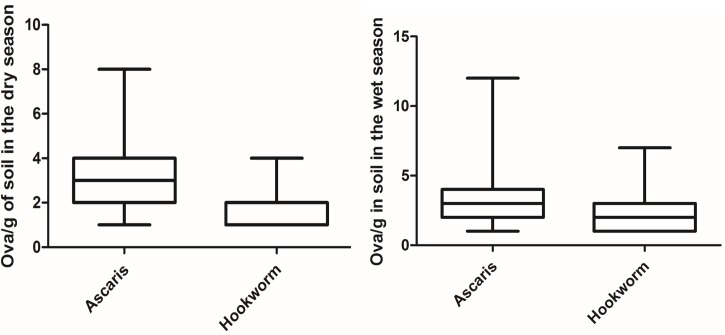
Distribution of *Ascaris* spp and hookworm ova in farm soil in the dry (n = 71) and wet (n = 107) seasons.

Ova of *Ascaris* spp, hookworm and *Schistosoma* spp (*Schistosoma* spp was found only in the irrigation water and is not further reported in this article) were identified in the irrigation water and soil in the vegetable farms. In general ova concentrations were higher in the wet season than the dry season for both irrigation water and soil samples (Refer to Table 1). Statistically there was difference in the concentration of hookworm ova in the two seasons and *Ascaris* spp concentration in the soil for between the seasons (Table 1). Figs 2 and 3 shows the distribution of the ova of *Ascaris* spp and hookworm in the irrigation water and soil for both the wet and dry seasons.

**Table 1:** Mean concentration (±S.D) of *Ascaris* spp and hookworm ova in irrigation water and soil for the dry and wet season in Kumasi, Ghana.

	Water			Soil		
	Wet season (n = 107)	Dry season (n = 78)	P value	Wet season (n = 107)	Dry season (n = 78)	P value
*Ascaris* spp (ova/L)	2.82 (±0.25)	2.62 (±0.13)	0.41	3.70 (±0.23)	2.90 (±0.21)	0.01
Hookworm (ova/L)	2.05 (±0.23)	1.38 (±0.10)	0.01	2.01(±0.16)	1.67 (±0.14)	0.16

### Prevalence of *Ascaris* spp and Hookworms among Farmers

**Fig 4:**
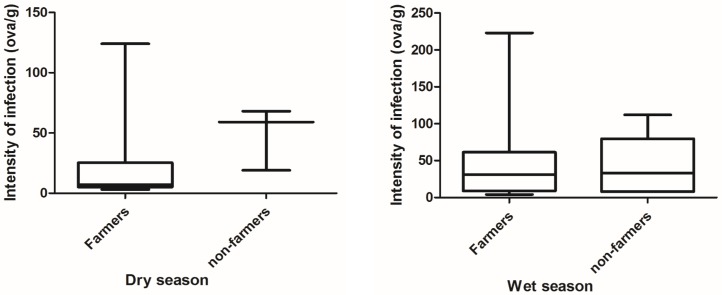
Distribution of *Ascaris* spp for farmers and non-farmers in both seasons.

**Fig 5:**
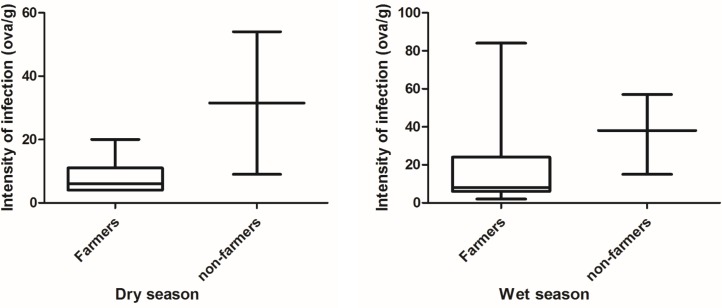
Distribution of hookworm infection intensity for farmers and non-farmers in both seasons.

Infection with the two parasites differed between seasons and between the farmers and the control group. The prevalence of *Ascaris* spp infection in the wet season was 15.77% (n = 165) for the farmers and 6.00% (n = 100) for the control group. Similarly, the prevalence of hookworm infection in the wet season was 12.73% (n = 165) for the farmers and 2.00% (n = 100) for the control group. In the dry season prevalence of *Ascaris* spp. was lower for both groups, the farmers had a prevalence of 11.02% (n = 127) and 5.74% (n = 52) for the control group. A much lower prevalence was recorded for hookworm infections for farmers with 4.72% (n = 127) however same prevalence as reported for *Ascaris spp* was reported for hookworm infections of the control group, but with different mean infections. Table 2 above shows the details of the range and significant difference and Figs 4 and 5 distribution of infection intensity.

**Table 2:** Range of eggs per gram (epg) of *Ascaris* spp and hookworm infection intensity in wastewater farmers and a control group in Kumasi, Ghana.

	Farmers			Non-farmers (Control group)		
	Wet season (n = 165)	Dry season (n = 127)	P value	Wet season (n = 100)	Dry season (n = 52)	P value
*Ascaris* spp (epg)	4–223	3–124	0.01	8–112	19–68	0.35
Hookworm (epg)	2–84	4–20	0.06	9–54	15–57	0.40

### Relationship between Parasite Ova in the Irrigation Water and Soil and the Incidence of Infection in Farmers

The concentration of the ova of the two reported helminths in both the irrigation water and soil and the intensity of infection of the exposed farmers showed a significantly positive relationship (p < 0.05) in the wet season (regression coefficient of 0.04; 95% CI: 0.203–0.69). The opposite was the case in the dry season (regression coefficient of -.0023; 95% CI: -.020 - .016), however this was not statistically significant (p > 0.05).

### Odds of Infection for Farmers and Non-Exposed Populations

The probability of farmers getting infected with STHs compared to the control group as a result of exposure to the ova in the irrigation water and the soil was higher in the wet season than in the dry season for the two STHs (Table 3). In the wet season, farmers exposed to irrigation water and soil were more likely than the control group to be infected with *Ascaris* spp (OR = 3.99, 95% CI: 1.15–13.86) and Hookworm (OR = 3.07, 95% CI: 0.87–10.82). However, there was lesser probability of infection in the dry season as shown in Table 3.

**Table 3:** Odds of infection with *Ascaris* spp and hookworm for farmers involved in wastewater irrigation compared with a control group in Kumasi, Ghana.

	DRY SEASON*	WET SEASON^$^
*Ascaris* spp	0.92 (95% CI; 0.33–2.56)	3.99 (95% CI; 1.15–13.86)
Hookworm	1.21 (95% CI; 0.24–6.20)	3.07 (95% CI; 0.87–10.82)

*In the dry season the study population was 127 for farmers and 52 for the control group

^$^In the wet season the study population was 165 for farmers and 100 for the control group

## Discussion

Helminth contamination of irrigation water is a serious public health issue, due to their persistence in the environment and their low infective dose. To safeguard human health, the WHO formulated guidelines for the use of wastewater in unrestricted agriculture [4], with a guideline value of <1 ova/L aimed at reducing the risk of infection. In this study, the mean concentration of STHs ova was higher than the recommended guideline value, especially for *Ascaris* spp (2.62 ova/L and 2.82 ova/L for dry and wet seasons, respectively) and hookworm (2.05 ova/L in the wet season), in line with similar results reported from studies in Ghana [3, 19, 20, 29] as well as other countries [25, 30, 35]. There was seasonal variation in the mean concentration of the STHs ova in the irrigation water, with the wet season showing higher concentrations than the dry season. Keraita *et al*. [26] reported similar patterns of helminth ova concentration in irrigation water from studies conducted in Kumasi. This occurrence could be attributed to rainfall and reduced temperatures which extend the survival period of the ova. However in general, helminth ova are resistant to many types of inactivation, with ova of *Ascaris* spp and *Taenia* spp having the highest resistance and survival rates [27, 28]. In addition to the lower temperatures and much lesser UV irradiation in the wet season, ova concentration could be increased during this period of the year due to run-offs from agricultural fields and other surrounding areas. Open defecation in areas close to these wastewater irrigated farms could also potentially lead to higher STHs ova concentrations after rainfalls. Wastewater irrigation does not only increase risk of STH infection due to exposure to the irrigation water but also exposure to the farm soil. Exposure to the farm soil in wastewater irrigated farms may result in higher risk of infection with STHs than risk attributable to the wastewater alone [29, 30]. This is due to a higher concentration of STHs ova in the soil as was seen in this study. Irrigation with wastewater result in accumulation of STHs ova in the soil, and therefore accounts for the higher concentration of ova. *A*. *lumbricoides* eggs have been found to attach to soil particles (especially clay) thereby contributing to their high concentrations in the soil samples [31]. In addition, contamination of soils could serve as a source for re-introduction of eggs into the irrigation water channels.

The elevated concentrations of STHs ova, above the WHO guideline levels, in the irrigation water and soil pose a risk of infection for farmers involved in the practice of wastewater irrigation. However, there is always a difference between the estimated risk and actual infections. The potential health risk is based on the number of pathogens in the wastewater or soil, while the actual health risk depends on an expansion of this concept, including: i) the period pathogens survive in water or soil; ii) the dose in which pathogens are infective to a human host and iii) host immunity for pathogens circulating in the environment [32]. The seasonal variation in STHs ova concentration in the irrigation water and soil was also apparent in the STHs infection intensity of the farmers, reflected in higher infection frequency in the wet season. There are other factors such as, climate, types of soils and hygiene behavior, which might have also contributed to this variation in infection rate [33]. The interrelationship with other confounding factors was seen with the correlation analysis where there was a weak association between the load or concentration in the irrigation water/soil and the intensity of the STH infection for the farmers, especially in the dry season.

To quantify the actual contribution of wastewater irrigation to STHs infection a control group of non-farmers who had no exposure to the irrigation water and soil but used same sanitation and portable water infrastructure as the farmers (staying in the same suburbs as the farmers) was needed. The increased probability of infection for farmers was expected due to a higher exposure to STHs ova over the course of the year as compared to the non-farmers. Infection with *Ascaris* spp and hookworm for the farmers is three times more likely than it is for non-farmers. This clearly indicates that wastewater use in irrigation contributes significantly to the incidence of helminthiases, as reported by many other studies [13, 29, 34, 35], especially in the wet season (Table 3). In the dry season the odds of infection for both farmers and non-farmers is not significantly different. This could be attributed to the lower concentrations of ova recorded in the irrigation water and soil during this time of the year.

It can be concluded from the results obtained in this study that exposure to STHs ova in irrigation water and soil contributes to infections in farmers and that farmers involved in the practice are three times likely to be infected with *Ascaris* spp and hookworm than unexposed populations. This is particularly so during the wet season where there is an increase in the concentration of the STHs ova. The results obtained show an epidemiological link between wastewater irrigation and helminth infection in Ghana, therefore emphasizing the need for regulations and interventions aimed at making the practice safer for the farmers which in turn would contribute significantly in breaking the cycle of infection.

## Supporting Information

S1 Checklist (DOC)

S1 Appendix Tables containing descriptive analysis of data. (DOCX)

S1 Data Tables Tables containing the raw data recorded for each sample of irrigation water, soil and stool. (XLSX)
