# Supplementary material for: Contribution of Wastewater Irrigation to Soil Transmitted Helminths Infection among Vegetable Farmers in Kumasi, Ghana
Source: PLoS Negl Trop Dis. 2016 Dec 6;10(12):e0005161. doi: 10.1371/journal.pntd.0005161 (PMC5140065; doi:10.1371/journal.pntd.0005161)
Supplement: S1 Appendix — (DOCX) [file pntd.0005161.s002.docx]

**DECRIPTIVE ANALYSIS OF DATA**

1. Descriptive Analysis of *Ascaris* spp and Hookworm ova Concentration Irrigation water and farm soil

Table S1: Descriptive analysis of *Ascaris* spp and Hookworm occurrence in farm soil in the wet season

|  | *Ascaris* spp | Hookworm |
| --- | --- | --- |
| Number of values | 107 | 107 |
|  |  |  |
| Minimum | 0 | 0 |
| 25% Percentile | 1 | 0 |
| Median | 3 | 1 |
| 75% Percentile | 4 | 2 |
| Maximum | 12 | 7 |
|  |  |  |
| Mean | 2.822 | 1.262 |
| Std. Deviation | 2.406 | 1.43 |
| Std. Error | 0.2326 | 0.1382 |
|  |  |  |
| Lower 95% CI of mean | 2.361 | 0.9877 |
| Upper 95% CI of mean | 3.284 | 1.536 |
|  |  |  |
| D'Agostino & Pearson omnibus normality test |  |  |
| K2 | 24.01 | 36.68 |
| P value | < 0.0001 | < 0.0001 |
| Passed normality test (alpha=0.05)? | No | No |
| P value summary | *** | *** |
|  |  |  |
| Coefficient of variation | 85.25% | 113.32% |
| Sum | 302 | 135 |

Table S2: Descriptive analysis of *Ascaris* spp and Hookworm occurrence in irrigation water in the wet season

|  | *Ascaris* spp | Hookworm |
| --- | --- | --- |
| Number of values | 107 | 107 |
|  |  |  |
| Minimum | 0 | 0 |
| 25% Percentile | 1 | 0 |
| Median | 2 | 0 |
| 75% Percentile | 3 | 1 |
| Maximum | 7 | 3 |
|  |  |  |
| Mean | 2.112 | 0.4393 |
| Std. Deviation | 1.5 | 0.7291 |
| Std. Error | 0.1451 | 0.07049 |
|  |  |  |
| Lower 95% CI of mean | 1.825 | 0.2995 |
| Upper 95% CI of mean | 2.4 | 0.579 |
|  |  |  |
| D'Agostino & Pearson omnibus normality test |  |  |
| K2 | 1.922 | 37.11 |
| P value | 0.3825 | < 0.0001 |
| Passed normality test (alpha=0.05)? | Yes | No |
| P value summary | ns | *** |
|  |  |  |
| Coefficient of variation | 71.04% | 166.00% |
|  |  |  |
| Skewness | 0.3175 | 1.629 |
| Kurtosis | -0.1485 | 2.013 |
|  |  |  |
| Sum | 226 | 47 |

Table S3: Descriptive analysis of *Ascaris* spp and Hookworm occurrence in farm soil in the dry season

|  | *Ascaris* spp | Hookworm |
| --- | --- | --- |
| Number of values | 71 | 71 |
|  |  |  |
| Minimum | 0 | 0 |
| 25% Percentile | 0 | 0 |
| Median | 2 | 1 |
| 75% Percentile | 3 | 1 |
| Maximum | 8 | 4 |
|  |  |  |
| Mean | 2.155 | 0.8451 |
| Std. Deviation | 1.849 | 1.023 |
| Std. Error | 0.2194 | 0.1214 |
|  |  |  |
| Lower 95% CI of mean | 1.717 | 0.6029 |
| Upper 95% CI of mean | 2.593 | 1.087 |
|  |  |  |
| D'Agostino & Pearson omnibus normality test |  |  |
| K2 | 4.934 | 11.83 |
| P value | 0.0848 | 0.0027 |
| Passed normality test (alpha=0.05)? | Yes | No |
| P value summary | ns | ** |
| Coefficient of variation | 85.80% | 121.09% |
| Skewness | 0.6454 | 1.061 |
| Kurtosis | -0.002419 | 0.3355 |
|  |  |  |
| Sum | 153 | 60 |

Table S4: Descriptive analysis of *Ascaris* spp and Hookworm occurrence in irrigation water in the dry season

|  | *Ascaris* spp | Hookworm |
| --- | --- | --- |
| Number of values | 71 | 71 |
|  |  |  |
| Minimum | 0 | 0 |
| 25% Percentile | 0 | 0 |
| Median | 2 | 1 |
| 75% Percentile | 3 | 2 |
| Maximum | 11 | 8 |
|  |  |  |
| Mean | 2.028 | 1.155 |
| Std. Deviation | 1.964 | 1.489 |
| Std. Error | 0.2331 | 0.1768 |
|  |  |  |
| Lower 95% CI of mean | 1.563 | 0.8024 |
| Upper 95% CI of mean | 2.493 | 1.507 |
|  |  |  |
| D'Agostino & Pearson omnibus normality test |  |  |
| K2 | 35.94 | 42.99 |
| P value | < 0.0001 | < 0.0001 |
| Passed normality test (alpha=0.05)? | No | No |
| P value summary | *** | *** |
|  |  |  |
| Coefficient of variation | 96.82% | 128.97% |
|  |  |  |
| Skewness | 1.637 | 1.995 |
| Kurtosis | 5.187 | 5.635 |
|  |  |  |
| Sum | 144 | 82 |

1. Descriptive Analysis of *Ascaris* spp and Hookworm infection of farmers and non-farmers (control group)

Table S5: Description of *Ascaris* spp and hookworm infection loads in farmers in the dry season

|  | *Ascaris* spp | Hookworm |
| --- | --- | --- |
| Number of values* | 14 | 6 |
|  |  |  |
| Minimum | 3 | 4 |
| 25% Percentile | 5 | 4 |
| Median | 7 | 6 |
| 75% Percentile | 25.25 | 11 |
| Maximum | 124 | 20 |
|  |  |  |
| Mean | 22.71 | 8 |
| Std. Deviation | 35.29 | 6.099 |
| Std. Error | 9.432 | 2.49 |
|  |  |  |
| Lower 95% CI of mean | 2.337 | 1.599 |
| Upper 95% CI of mean | 43.09 | 14.4 |
|  |  |  |
| KS normality test |  |  |
| KS distance | 0.3655 | 0.3333 |
| P value | < 0.0001 | 0.0359 |
| Passed normality test (alpha=0.05)? | No | No |
| P value summary | *** | * |
|  |  |  |
| D'Agostino & Pearson omnibus normality test |  |  |
| K2 | 18.82 | N too small |
| P value | < 0.0001 |  |
| Passed normality test (alpha=0.05)? | No |  |
| P value summary | *** |  |
|  |  |  |
| Coefficient of variation | 155.38% | 76.24% |
|  |  |  |
| Geometric mean | 10.64 | 6.689 |
| Lower 95% CI of geo. mean | 5.495 | 3.534 |
| Upper 95% CI of geo. mean | 20.61 | 12.66 |
|  |  |  |
| Skewness | 2.337 | 2.079 |
| Kurtosis | 5.168 | 4.54 |
|  |  |  |
| Sum | 318 | 48 |

* A total of 127 farmers were studied with 14 and 6 infected with *Ascaris* spp and hookworm respectively

Table S6: Description of *Ascaris* spp and hookworm infection loads in farmers in the wet season

|  | *Ascaris* spp | Hookworm |
| --- | --- | --- |
| Number of values* | 26 | 21 |
|  |  |  |
| Minimum | 4 | 2 |
| 25% Percentile | 8.75 | 6 |
| Median | 31 | 8 |
| 75% Percentile | 61.25 | 24 |
| Maximum | 223 | 84 |
|  |  |  |
| Mean | 47.96 | 17.86 |
| Std. Deviation | 58.28 | 21.18 |
| Std. Error | 11.43 | 4.622 |
|  |  |  |
| Lower 95% CI of mean | 24.42 | 8.216 |
| Upper 95% CI of mean | 71.5 | 27.5 |
|  |  |  |
| KS normality test |  |  |
| KS distance | 0.2253 | 0.2865 |
| P value | 0.0015 | < 0.0001 |
| Passed normality test (alpha=0.05)? | No | No |
| P value summary | ** | *** |
|  |  |  |
| D'Agostino & Pearson omnibus normality test |  |  |
| K2 | 19.12 | 19.97 |
| P value | < 0.0001 | < 0.0001 |
| Passed normality test (alpha=0.05)? | No | No |
| P value summary | *** | *** |
|  |  |  |
| Coefficient of variation | 121.51% | 118.61% |
|  |  |  |
| Geometric mean | 24.57 | 11.09 |
| Lower 95% CI of geo. mean | 15.09 | 7.224 |
| Upper 95% CI of geo. mean | 40.01 | 17.03 |
|  |  |  |
| Skewness | 1.944 | 2.159 |
| Kurtosis | 3.564 | 4.34 |
|  |  |  |
| Sum | 1247 | 375 |

*A total of 165 farmers were studied with 26 and 21 infected with *Ascaris* spp and hookworm respectively

Table S7: Description of *Ascaris* spp and hookworm infection loads in the control group in the wet season

|  | *Ascaris* spp | Hookworm |
| --- | --- | --- |
| Number of values* | 6 | 2 |
|  |  |  |
| Minimum | 8 | 9 |
| 25% Percentile | 8 | 9 |
| Median | 33 | 31.5 |
| 75% Percentile | 79.75 | 54 |
| Maximum | 112 | 54 |
|  |  |  |
| Mean | 43.83 | 31.5 |
| Std. Deviation | 42.98 | 31.82 |
| Std. Error | 17.55 | 22.5 |
|  |  |  |
| Lower 95% CI of mean | -1.268 | -254.4 |
| Upper 95% CI of mean | 88.93 | 317.4 |
|  |  |  |
| KS normality test |  |  |
| KS distance | 0.2912 | 0.2602 |
| P value | > 0.10 | > 0.10 |
| Passed normality test (alpha=0.05)? | Yes | Yes |
| P value summary | ns | ns |
|  |  |  |
| D'Agostino & Pearson omnibus normality test |  |  |
| K2 | N too small | N too small |
| P value |  |  |
| Passed normality test (alpha=0.05)? |  |  |
| P value summary |  |  |
|  |  |  |
| Coefficient of variation | 98.04% | 101.02% |
|  |  |  |
| Geometric mean | 25.16 | 22.05 |
| Lower 95% CI of geo. mean | 6.899 | 0.000251 |
| Upper 95% CI of geo. mean | 91.77 | 1.94E+06 |
|  |  |  |
| Skewness | 0.7585 |  |
| Kurtosis | -0.7521 |  |
|  |  |  |
| Sum | 263 | 63 |

*A total of 100 participants were studied with 6 and 2 infected with *Ascaris* spp and hookworm respectively

Table S8: Description of *Ascaris* spp and hookworm infection loads in the control group in the dry season

|  | *Ascaris* spp | Hookworm |
| --- | --- | --- |
| Number of values* | 3 | 3 |
|  |  |  |
| Minimum | 19 | 15 |
| 25% Percentile | 19 | 15 |
| Median | 59 | 38 |
| 75% Percentile | 68 | 57 |
| Maximum | 68 | 57 |
|  |  |  |
| Mean | 48.67 | 36.67 |
| Std. Deviation | 26.08 | 21.03 |
| Std. Error | 15.06 | 12.14 |
|  |  |  |
| Lower 95% CI of mean | -16.13 | -15.58 |
| Upper 95% CI of mean | 113.5 | 88.91 |
|  |  |  |
| KS normality test |  |  |
| KS distance | 0.3207 | 0.1919 |
| P value | > 0.10 | > 0.10 |
| Passed normality test (alpha=0.05)? | Yes | Yes |
| P value summary | ns | ns |
|  |  |  |
| D'Agostino & Pearson omnibus normality test |  |  |
| K2 | N too small | N too small |
| P value |  |  |
| Passed normality test (alpha=0.05)? |  |  |
| P value summary |  |  |
|  |  |  |
| Coefficient of variation | 53.60% | 57.36% |
|  |  |  |
| Geometric mean | 42.4 | 31.91 |
| Lower 95% CI of geo. mean | 7.472 | 5.827 |
| Upper 95% CI of geo. mean | 240.6 | 174.7 |
|  |  |  |
| Skewness | -1.503 | -0.2841 |
| Kurtosis |  |  |
|  |  |  |
| Sum | 146 | 110 |

*A total of 52 participants were studied with 3 and 3 infected with *Ascaris* spp and hookworm respectively

3.0: Regression results of infection intensity and concentration of STHs ova

Table S9: Poisson regression between Infection loads and concentration of STHs ova in both irrigation water and soil in the dry season

Poisson regression Number of obs = 38

LR chi2(1) = 0.06

Prob > chi2 = 0.8018

Log likelihood = -978.74699 Pseudo R2 = 0.0000

----------------------------------------------------------------------------------

Infectionloads ds | Coef. Std. Err. z P>|z| [95% Conf. Interval]

-----------------+----------------------------------------------------------------

wastewatersoilds | -.0023121 .0092193 -0.25 0.802 -.0203817 .0157575

_cons | 3.755762 .0623269 60.26 0.000 3.633603 3.87792

Table S10: Poisson regression between Infection loads and concentration of STHs ova in both irrigation water and soil in the wet season

Poisson regression Number of obs = 16

LR chi2(1) = 12.27

Prob > chi2 = 0.0005

Log likelihood = -287.39477 Pseudo R2 = 0.0209

------------------------------------------------------------------------------

B | Coef. Std. Err. z P>|z| [95% Conf. Interval]

------------+----------------------------------------------------------------

C | .0449809 .0125891 3.57 0.000 .0203067 .0696551

_cons| 2.748494 .1172516 23.44 0.000 2.518685 2.978303

------------------------------------------------------------------------------
